# Biomimetic bone scaffold functionalized with traditional Chinese medicine ingredients and collagen-binding IGF-1 for bone regeneration

**DOI:** 10.1007/s10856-026-07041-2

**Published:** 2026-04-06

**Authors:** Xiaobo Gao, Bocheng Lei, Xiaomin Zhao, Ying Zhang

**Affiliations:** 1 Department of Stomatology, Chifeng municipal hospital, Chifeng, PR China; 2 Affiliated Chifeng Clinical Medical College of Inner Mongolia Medical University, Chifeng, PR China; 3 Stomatology College of Chifeng University, Chifeng, PR China

## Abstract

Many active ingredients from traditional Chinese medicine (TCM) have been applied in bone tissue engineering. However, the fuzzy mechanism of these ingredients limits their application. In this study, a novel biomimetic bone scaffold functionalized with two TCM ingredients, nano deer bone meals (nBM) and neferine (Nef), as well as collagen-binding IGF-1 (CBD-IGF-1) was fabricated via a combination of phase inversion and particle leaching methods. The biomimetic CIGF1&Nef@PLGA/nBM scaffold mimicked cancellous bone in terms of its porous structure and bone matrix composition rich in collagen. Both Nef contained in the scaffold and CBD-IGF-1 bound to collagen matrix could be slowly released from the scaffold, exerting long-term and safe bioactivity. In vitro tests showed that the CIGF1&Nef@PLGA/nBM composite significantly enhanced the adhesion, proliferation and osteogenic differentiation of MC3T3-E1 cells on the material. Nef and IGF-1 in CIGF1&Nef@PLGA/nBM scaffold exerted a synergistic effect on IGF-1-mediated osteogenic differentiation via IGF-1R axis to ensure the osteogenic activity of the biomimetic scaffold. In vivo skull defect repair experiment confirmed that CIGF1&Nef@PLGA/nBM could promote the neocortical bone formation and osseous maturation, further accelerating the bone defect repair. The findings of this research demonstrated that the biomimetic CIGF1&Nef@PLGA/nBM scaffold is a promising implantation in bone defect therapy.

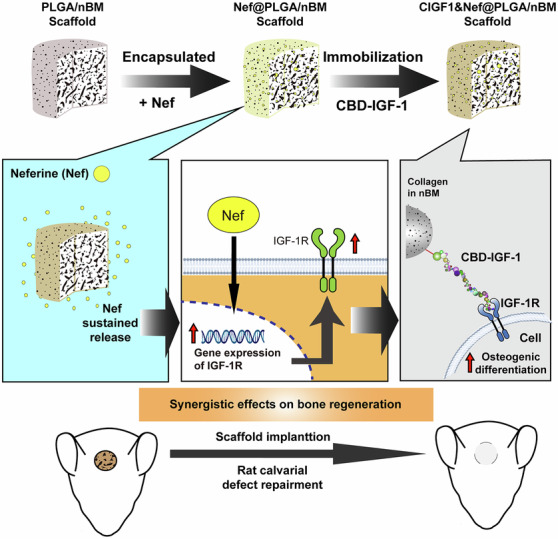

## Introduction

Large bone defects represent a significant clinical challenge. Among various treatments, autologous bone grafting has been regarded as the gold standard for the repair of large bone defects [1, 2]. However, although autologous bone grafting achieves remarkable therapeutic effects, it is associated with limitations, such as donor site complications, limited donor bone availability, and an increased risk of infection. In recent years, bone tissue engineering has emerged as a promising therapy for the treatment of large bone defects, circumventing the drawbacks of autologous bone grafting. Bone tissue engineering involves filling the defect site with scaffolds to provide support for bone repair and introducing osteogenic active ingredients (such as growth factors, natural medicines, etc.) to promote bone regeneration [3, 4]. The success of bone tissue engineering largely depends on the selection of scaffold materials and the regulation of active ingredients loaded into scaffolds or presented in the defect healing microenvironment. As a potential active ingredient that can be applied in bone tissue engineering, traditional Chinese medicine (TCM) has been applied as an effective means to promote the repair of bone tissue damage for over 2000 years. Many TCM formulas are empirically believed to be effective in treating bone injuries [5–7]. However, the active ingredients in these formulas are not clear, which hinders the application of TCM in bone repair. Nowadays, the concept of integrating TCM with western medicine has promoted research related to Chinese medicines. More and more active ingredients with clear therapeutic effects on bone tissue injury repair have been identified and extracted [7]. However, application of ingredients to bone tissue engineering was rarely reported.

Currently, bone-derived products are widely used as bone repair materials. Bones from Sika deer and red deer are rich in active proteins, collagen, trace elements, and other beneficial components. In TCM, deer bone is revered for its ability to fortify tendons and bones [8]. Relevant research has illuminated that polypeptide extracts derived from Sika Deer bones can ameliorate the microscopic structural abnormalities associated with osteoporosis and stimulate the expression of eNOS in osteoblasts. Xue et al. conducted a study on the impact of a compound deer bone extract on osteoporosis, revealing that it could alleviate the condition by effectively augmenting the number and thickness of tibial trabeculae, ensuring their tight interconnections [9]. An et al. administered deer bone polypeptide orally to rats with glucocorticoid-induced osteoporosis [10]. Their findings demonstrated that deer bone polypeptide could counteract the dexamethasone-induced imbalance in calcium and phosphorus metabolism, decrease alkaline phosphatase levels, elevate osteocalcin levels, inhibit bone resorption, promote bone formation, and ameliorate the pathological changes and microscopic structure of bone in these osteoporotic rats. In Wu’s report, a bone tissue engineering microsphere containing nano deer bone powder (nBM) was developed. Experimental results confirmed that the addition of nBM significantly enhanced the osteogenic activity of the bone tissue engineering microspheres [11]. These studies underscore the potential of deer bone as a biomimetic matrix component for bone tissue engineering repair. By integrating diverse deer bone products, including deer bone powder, polypeptides, and demineralized extracellular matrix (ECM), with biocompatible polymer materials, it is conceivable to address the limitation of poor biological activity inherent in polymer materials and enhance the repair efficacy of the composite materials.

Various signal molecules, including TCM ingredients and growth factors, can promote osteoblast proliferation, differentiation and maturation via different signaling pathway, functioning eminently in bone regeneration [12]. Neferine (Nef), a natural dibenzyl isoquinoline alkaloid derived from lotus seeds, has been used in TCM for over a millennium to alleviate inflammation. Modern medical research has further revealed its multifaceted benefits. Nef has demonstrated efficacy in moderating bleomycin-induced pulmonary fibrosis, inhibiting the proliferation of vascular smooth muscle cells, and decreasing platelet aggregation rates. Additionally, it exhibits therapeutic potential in managing arrhythmias [13, 14]. In addition, Nef is also believed to be involved in the regulation of insulin-like growth factor 1 (IGF-1) during the injury healing process, influencing tissue damage and the microenvironment. In the context of cardiomyocyte health, Nef has been shown to regulate the IGF-1R/Nrf2 signaling pathway in H9c2 cardiomyocytes exposed to doxorubicin. Doxorubicin typically triggers a surge in mitochondrial superoxide levels in these cells, diminishes their antioxidant capacity, and impedes the activation of the IGF-1R signaling pathway by inhibiting the PI3K/Akt/mTOR cascade. Nef counteracts these effects by activating the IGF-1R signaling pathway, enhancing cellular antioxidant capacity, upregulating PI3K/Akt/mTOR expression, and significantly restraining mitochondrial superoxide production and autophagy [15]. Recently, Nef has emerged as a promising therapeutic candidate for addressing osteolytic bone conditions, such as osteoporosis. Chen et al. reported that Nef inhibits RANKL-induced osteoclast formation. Furthermore, it effectively curtails the bone resorption activity of mature osteoclasts. Mechanistically, Nef achieves this by inhibiting RANKL-induced activation of the NF-κB signaling pathway, which subsequently downregulates the expression of NFATC1, leading to the downregulation of related osteoclast marker genes. Concurrently, Nef promotes the differentiation and bone mineralization activity of MC3T3-E1 cells [16]. Based on the aforementioned research, Wu et al. prepared a bone tissue engineering scaffold containing Nef for bone injury repair. The composite scaffold containing Nef was first shown to effectively activate the IGF-1R/PI3K/AKT/mTOR pathway, thereby enhancing IGF-1-mediated osteogenic differentiation [17]. These findings underscore the potential of Nef as a signaling factor in bone tissue engineering.

In the research on osteoblast genesis promotion of Nef, IGF-1 was proposed as a key growth factor, suggesting a potential synergistic effect between Nef and IGF-1. [17] IGF-1 is a growth factor that exhibits prominent effects in bone injury repair, promoting both the proliferation of osteoblasts and osteogenic differentiation. Unfortunately, like other growth factors, IGF-1 has a short half-life and is prone to being attenuated with body fluid, which weakens its therapeutic efficacy in vivo. To overcome these limitations, the development of bioengineered drug delivery systems capable of stably loading and releasing IGF-1 has attracted a lot of interest [18, 19]. Growth factors equipped with binding tags provided through recombinant expression and ligation techniques demonstrate significant potential in tissue engineering applications. The binding domains derived from antibodies, enzymes, and other biomacromolecules play a crucial role in the integration of growth factors with biomaterials [20]. According to recent reports, the growth factors with collagen-binding domain (CBD) have been lucubrated as effective agents binding specially to collagen material which actively governs regeneration of bone fracture. In Saito’s study, an allogenic demineralized bone powder (DBP) with basic fibroblast-derived growth factor containing a polycystic kidney disease (PKD) domain and CBD from Clostridium histolyticum collagenase (ColH) was developed. and the stimulatory effects of bFGF-PKD-CBD combined with allogenic DBP on bone growth in a mouse femur fracture model were investigated. bFGF-PKD-CBD/DBP composite accelerates callus formation in a bone fracture model in mice and clearly showed that the composite also increases bone mineral density at fracture sites compared to bFGF/DBP [21]. In addition, bFGF-PKD-CBD/DBP increased callus volume and bone mineral content to similar levels in fractures treated with a tenfold higher amount of bFGF. In Liu’s report, CBD was conjugated to bone morphogenetic protein-2 mimetic peptide (BMP2-MP) to prepare collagen-binding BMP2-MP (CBD-BMP2-MP), then, CBD-BMP2-MP was combined with PLGA/collagen (PLGA/COL) composite scaffold for the surface modification of scaffold. Compared with BMP2-MP, CBD-BMP2-MP had better combine capacity on PLGA/COL scaffold. And the CBD-BMP2-MP modified 3D porous PLGA/COL scaffold has good biocompatibility and osteogenic activity [22]. The growth factors with CBD are widely investigated in bone tissue engineering. However, studies on IGF-1 with CBD in bone repair are relatively rare.

In the present research, nBM and Nef were incorporated into a PLGA substrate to fabricate a porous biomimetic bone tissue engineering scaffold (Nef@nBM/PLGA) via a combination of phase inversion and particle leaching methods. Then, the CBD-IGF-1 was first used as an osteogenesis-promoting active ingredient and a synergistic partner of Nef, bound to the collagen in nBM, for the preparation of a novel bone tissue engineering scaffold (CIGF1&Nef@nBM/PLGA). The biocompatibility and osteogenic activity of the CIGF1&Nef@nBM/PLGA scaffold were studied in vitro. Subsequently, the ability of the CIGF1&Nef@nBM/PLGA scaffold to promote bone defect repair was evaluated using a rat cranial defect repair model. This research will provide a new research direction for the application of TCM ingredients in bone tissue engineering.

## Materials and Methods

### Materials

PLGA (molecular Weight=200,000, LA/GA = 75/25) was obtained from the Jinan Daigang Biomaterial Co., Ltd. The fresh sika deer leg bones used in the experiments were fresh samples taken within 24 h after slaughter, which were provided by the breeding and slaughtering plant in Deer Town, Changchun City, Jilin Province. Nano deer bone meal (nDB) obtained by autoclaving and ball milling was provided by Department of Orthopedics, China-Japan Union Hospital of Jilin University. The biosafety has been confirmed in previous reports [23, 24]. The CBD-IGF-1 (amino acid sequence: TKKTLRTGGG GGGPETLCGA ELVDALQFVC GDRGFYFNKP TGYGSSSRRA PQTGIVDECC FRCDLRRLEM YCAPLKPAKS A) was provided and authenticated by Changchun Ubbiotech Biotechnology Co., Ltd. The expression level was determined based on a 1 L expression system and calculated using the BCA assay. Purity was analyzed by ImageJ according to the results of SDS‑PAGE result. The reagents for the experiments were purchased from Sigma-Aldrich (USA) if not otherwise noted.

### Fabrication of the composite scaffolds

Nef@nBM/PLGA composite was obtained via phase inversion and particle leaching methods. Briefly, nBM and Nef were uniformly dispersed in the PLGA solution in NMP (20%, w/v) by stirring. The solution and sodium chloride particles (with a particle size range of 250 ~ 400 μm) were then mixed in a cylindrical mold at a volume ratio of 1:9 and compacted. The mixture was then frozen at −80 °C for 12 h. Afterwards, the frozen scaffold was placed in deionized water. The water was changed every 6 h for 3 days. After the NMP solvent and sodium chloride particles were completely removed, the PLGA/PAP scaffold was freeze-dried for 12 h and stored in a drying oven. In order to obtain the CIGF1&Nef@nBM/PLGA, the Nef@nBM/PLGA scaffold was immersed in CBD-IGF-1 solution (pH 7.8) at a concentration of 100 ng/mL at 4 °C for 2 h. The scaffold was then washed with PBS (pH 7.0) to remove free proteins.

### Characterization of the composite scaffolds

The micromorphology and microstructure of scaffolds was observed by ESEM (XL30 FEG, Philips) and micro-CT (Bruker, SkyScan1172, Germany). The porosity of the scaffolds was computed using CTAn software based on the micro-CT data.

### Detection of Nef release efficiency

The quantification of Nef concentration was performed through UV spectrophotometric analysis at a detection wavelength of 270 nm. A standard calibration curve was initially established using methanolic solutions containing known Nef concentrations (Fig. S2). To determine the encapsulation efficiency (EE) and drug loading capacity (LC), precisely weighed scaffolds (100 mg) were subjected to mechanical homogenization followed by methanol extraction (1 mL) to solubilize the incorporated Nef. The resulting suspension was centrifuged at 10,000 × g for 10 min to isolate the drug-containing supernatant. The residual Nef mass (designated as M2) in the supernatant was subsequently quantified using the pre-established standard curve, while M1 represented the initial drug amount in the polymer solution prior to scaffold fabrication. All experimental measurements were conducted in triplicate to ensure data reproducibility. *EE* and *LC* of the Nef were respectively calculated according to formula(1) and (2):

1 $${EE}=M2/M1\times 100 \%$$

2 $${LC}=M2/100{mg}\times 100 \%$$

The in vitro release profile of Nef was evaluated under simulated physiological conditions (37 °C, PBS pH 7.4). Scaffolds (100 mg) were incubated in 20 mL release medium with continuous agitation (100 rpm). At predetermined intervals (0.5–168 h), 1 mL aliquots were withdrawn and immediately replaced with equal volumes of pre-warmed fresh PBS to maintain sink conditions. The sampled solutions underwent liquid-liquid extraction with ethyl acetate (3 × 1 mL) after vortex-mixing for 30 s. The combined organic phases were concentrated under reduced pressure (40 °C, 0.1 MPa) using a rotary evaporator (Büchi R-300) and reconstituted in 1 mL methanol through ultrasonic dispersion (40 kHz, 5 min). The cumulative released drug mass (denoted as m) was quantified via the established calibration curve. This release kinetic study was systematically conducted with triplicate experiments to ensure methodological reliability.

The percentage of released Nef (*RP*) was calculated as formula (3):

3 $${RP}=\left(m1+\ldots \ldots {mn}\right)/M2\times 100 \%$$

### Adhesion and stability of CBD-IGF-1

The protein conjugation efficiency was evaluated through optimized ligand immobilization protocols. Scaffolds were immobilized in 24-well culture plates and loaded with 1 mL CBD-IGF-1 solution (100 ng/mL in PBS, pH 7.4) for prolonged incubation (4 °C, 12 h). The primary supernatant was collected after centrifugation (400 × g, 5 min) and designated as S1. Subsequently, three successive wash cycles were performed using 1 mL PBS-T buffer (0.1% Tween 20, pH 7.2) under orbital shaking (150 rpm, 5 min/cycle) to remove non-specifically adsorbed proteins. The secondary through quaternary wash fractions (S2-S4) were systematically collected following each washing phase. Quantitative analysis of IGF-1 content in all fractions was conducted using a commercially available ELIZA kit (COIBO BIO, Shanghai) according to the manufacturer’s standardized protocol, with absorbance measurements performed at 450 nm using a microplate reader. The standard calibration curve of the IGF-1 ELIZA kit was shown in Fig. S3. The adhesion amount of fusion protein was calculated using the following formula (4):

4 $${Adhesion}\,{Amount}({AA})={Initial}\,{Amount}({IA})-{Sample}\,{Amount}({SA})$$

*IA* was the total protein amount in solution. *SA* was the total amount of unconjugated protein on the microspheres (*SA* = *S1* + *S2* + *S3* + *S4*).

The dynamic release kinetics of IGF-1 were quantitatively assessed using a modified sample-and-replace protocol under physiological simulation conditions (37 °C, 100 rpm). Scaffolds (100 mg) were loaded into 24-well plates containing 1 mL PBS (pH 7.2) maintained at 37 °C using a thermostatic shaker (TS-100C, Biosan). At predetermined intervals (10 min -72 h), 200 μL of supernatant was collected for analysis and replaced with fresh PBS (pH 7.2). The stability of IGF-1 is determined by the percentage of the protein retained on the scaffold to the total adhesion protein. Commercial IGF-1 was used as a control.

### Cell culture

MC3T3-E1 cells (Shanghai Institutes for Biological Sciences, Chinese Academy of Sciences) was used in this research. The cells were expanded in Dulbecco’s minimum essential medium (DMEM, Corning, USA) containing 10% FBS (Gibco, USA), 100 mg/L streptomycin (Sigma) and 63 mg/L penicillin (Sigma).

### Morphology and proliferation of MC3T3-E1 cells on the scaffolds

The osteogenic potential of scaffolds was systematically evaluated through quantitative proliferation analysis and cyto-morphology characterization. Following UV sterilization (30 min), scaffolds were immobilized in 24-well culture plates. MC3T3-E1 cells were seeded at a standardized density of 3×10⁴ cells/well in DMEM supplemented with 10% FBS. Cellular proliferation kinetics were assessed at 1-, 3-, and 7-day intervals using CCK-8 assay (Dojindo Laboratories). At each evaluation timepoint, culture medium was replaced with serum-free medium containing 10% CCK-8 reagent (v/v) for 2 h incubation under humidified atmosphere (37 °C, 5% CO₂). Subsequently, 200 μL aliquots were transferred to 96-well plates for optical density measurement at 450 nm using a Multiskan SkyHigh microplate reader (Thermo Fisher Scientific, USA).

For cyto-morphology analysis, cells were fixed with 4% paraformaldehyde in PBS (pH 7.4) after 72 h cultivation, followed by three successive PBS washes to remove fixation residuals. Membrane-permeabilized cells (0.1% Triton X-100, 10 min) were stained with 2 μM Calcein-AM (Beyotime Biotechnology) in Hanks’ Balanced Salt Solution for 30 min at 37 °C. Fluorescent images of cellular morphologies were captured using a fluorescence microscope (Nikon, E80i, Japan) with 488 nm excitation/515 nm emission parameters. Quantitative adhesion assessment was performed by measuring fluorescence area ratio using ImageJ.

### Osteogenic differentiation of MC3T3-E1 cells on the scaffolds

The osteogenic differentiation potential of MC3T3-E1 cells cultured on scaffolds was systematically evaluated through alkaline phosphatase (ALP) activity profiling over 7-day cultivation periods. Following designated culture intervals, cell-scaffold constructs were fixed with 4% paraformaldehyde in phosphate-buffered saline (PBS, pH 7.4) at 4 °C for 30 min, followed by three sequential PBS washes (5 min each) to remove residual fixative. ALP staining was performed using an NBT/BCIP chromogenic substrate kit (Beyotime Biotechnology, China) under optimized conditions (37 °C, 2 h in dark), with subsequent imaging conducted via microscope. For quantitative analysis, cells were washed with ice-cold PBS and lysed using RIPA buffer supplemented with protease inhibitors (1:100 v/v), followed by three freeze-thaw cycles (−80 °C/25 °C alternation). Clarified supernatants obtained through centrifugation (12,000 × g, 10 min, 4 °C) were subjected to enzymatic activity quantification using p-nitrophenyl phosphate (pNPP) substrate (Solarbio, China) with kinetic measurements at 405 nm. Total protein content normalization was achieved via bicinchoninic acid (BCA) assay (Pierce™). The relative ALP activity was calculated as formula (5):

5 $${Relative}\,{ALP}\,{activity}={ALP}({{OD}}_{405})/{BCA}({{OD}}_{562})$$

### Calcium deposition

Calcium deposition in MC3T3-E1 cells at 14 days was observed via alizarin red S (ARS) staining. Cells were fixed with 4% paraformaldehyde at 4 °C for 30 min and subsequently subjected to ARS staining using a standardized protocol. Scaffolds were incubated with 1% ARS solution (pH 4.2, Solarbio) in dark humidified conditions (37 °C, 30 min) to ensure specific calcium-phosphate complex binding. Finally, the ARS-stained cells were observed via optical microscopy. For quantitative spectrophotometric analysis, ARS-calcium complexes were solubilized through 30 min orbital shaking (150 rpm) in 10% cetylpyridinium chloride (CPC, Aladdin Biochemical) at ambient temperature. The resultant chromogenic solution was centrifuged (12,000 × g, 10 min, 4 °C) to remove particulate interference, and 200 μL aliquots were transferred in technical triplicate to 96-well plates. The absorbance of the solution was read at 540 nm via a multifunctional microplate scanner.

### Real-time polymerase chain reaction

After 7 days of culture, the expression of four genes, collagen-I (COL1), runt-related transcription factor 2 (Runx2), osteopontin (OPN), and IGF-1R was quantified via real-time polymerase chain reaction (RT-PCR). The cells were digested from different scaffolds, and the total RNA from each group was extracted with TRIzol reagent according to the reagent instructions. The total RNA concentration and purity were determined with a NanoDrop spectrophotometer (Tecan M200). The RNA samples were reverse transcribed into cDNA via a PrimeScript kit (Takara, China) for RT‒PCR. The RT‒PCR amplification procedure was performed as follows: initial denaturation at 95 °C for 10 min, followed by 40 cycles of 95 °C or 30 s, 58 °C for 1 min, and 72 °C for 1 min. The sequence of RT-PCR primers designed by Beacon Designer8.14 is shown in Table S1. These primers were synthesized by Co., Ltd. GAPDH was used as an internal reference gene.

### Immunofluorescence staining

Immunofluorescence staining was performed on the cell samples to determine the osteogenic differentiation of cells. The cells were fixed with 4% paraformaldehyde and then treated with 1% Triton X-100. The cells were blocked with 10% sheep serum and incubated with diluted Runx2, IGF-1R primary antibodies at 4 °C overnight. Information of primary antibodies was listed in Table S2. After the cells were washed with PBS, fluorescent secondary antibodies were added. Finally, the cells were observed under a fluorescence microscope. Measure the mean fluorescence intensity using ImageJ (NIH, USA).

### Animals and experimental protocol

Ten female Wistar rats (8 weeks, 200–240 g) obtained from Vital River Laboratory Animal Technology Co., Ltd. (Beijing, China) were acclimatized under specific pathogen-free conditions for 7 days prior to experimentation, with all procedures conducted in strict compliance with ARRIVE guidelines and approved by the Animal Ethics Committee of chifeng municipal hospital. Surgical interventions were performed under deep anesthesia induced by intraperitoneal injection of 3% pentobarbital sodium (30 mg/kg), with anesthetic depth confirmed by loss of pedal withdrawal reflex. Following dorsal cranium shaving and sequential disinfection using povidone-iodine (10% w/v) and ethanol (75% v/v), a 20 mm mid-sagittal incision was created posteriorly from the lambda landmark using sterile surgical blades, exposing the parietal bones through blunt dissection of subcutaneous fascia. Bilateral full-thickness circular cranial defects (Ø5.0 mm) were surgically created using a trephine bur (Dentsply Sirona) at 800 rpm under continuous saline irrigation, maintaining dural integrity. The resultant 20 defects were randomized into five experimental groups (*n* = 4 defects/group) through a block design, including sham controls (non-implantation) and four biomaterial-implanted groups, with intra-group specimens distributed across different animals to control inter-individual variability. Postoperative management encompassed intramuscular administration of benzylpenicillin sodium (80,000 IU/day, North China Pharmaceutical) for 5 days, combined with sustained-release buprenorphine analgesia (1.0 mg/kg, s.c.)

Eight weeks after surgery, all the animals were sacrificed. Micro-CT (ScanScan v1172, Bruker) was performed to observe the bone defects. The regeneration coverage rate was detected by ImageJ. Bone volume fraction (BVF, BV/TV) at the defect site was detected by CTAn. The final repair effect was evaluated by tissue section staining (H&E and Masson).

### Statistical analysis

All the data are shown as the means ± standard deviations. One-way analysis of variance with a post hoc test was used to determine significant differences. The data presented in this study are presented as the means ± standard deviations. *p < 0.05 was considered to indicate statistical significance. All the quantitative data were analyzed via Origin 8.0 software (Origin Lab Corporation, USA).

## Results

### Optimization of the amount of nBM in the scaffolds

**Fig. 1:**
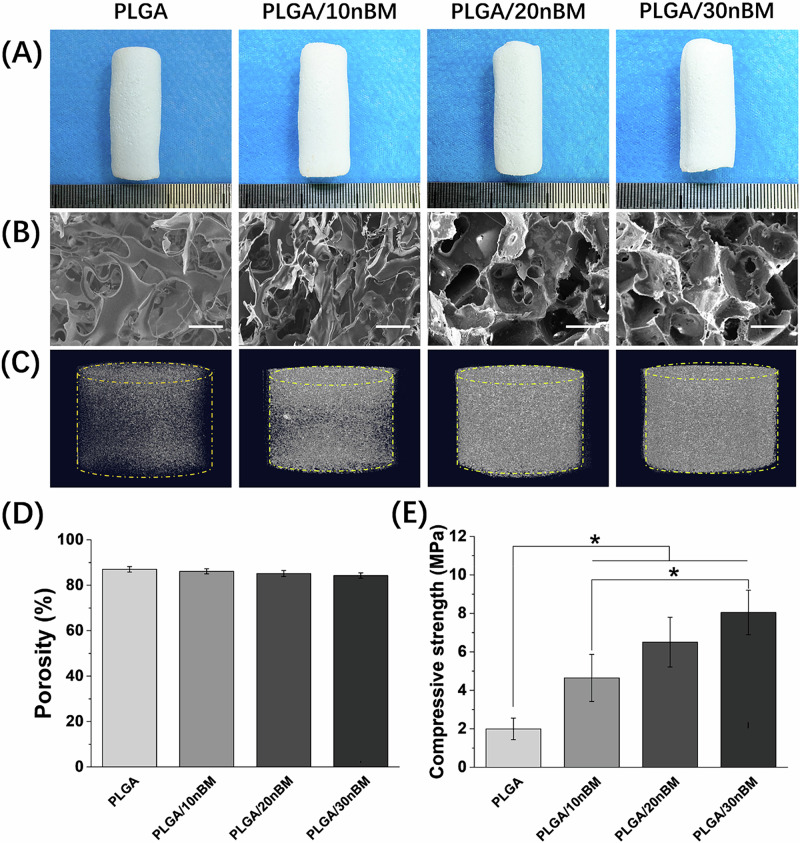
Characterization of scaffolds with different nBM content. **A** the appearance of each scaffold. **B** the representative SEM photos of the fractured surfaces of different scaffolds. **C** the 3D micro-CT reconstructions of different scaffolds. **D** quantitative analysis of porosity of different scaffolds. **E** the compressive strength of different scaffolds. Bar = 200 μm, *n* = 3, *p < 0.05

In this study, composite scaffolds incorporating varying percentages of nBM at 10%, 20%, and 30% weight ratios, designated as PLGA/10nBM, PLGA/20nBM, and PLGA/30nBM respectively, were fabricated to systematically evaluate the optimal nBM incorporation level. As illustrated in Fig. 1A, all scaffolds successfully replicated the cylindrical geometry of the mold. The scaffolds exhibited a uniform white coloration characteristic of the base materials (PLGA and nBM). SEM analysis of fractured surfaces (Fig. 1B) and cross-sectional X-ray images (Fig. S4) revealed an interconnected porous architecture across all groups, with predominant pore dimensions ranging 250–400 μm. PLGA and PLGA/10nBM scaffolds displayed irregularly contracted pore geometries, whereas PLGA/20nBM and PLGA/30nBM maintained more defined angular pore contours. This phenomenon suggested that increasing nBM content might mitigate structural deformation during lyophilization through enhancing mechanical stability. 3D micro-CT reconstructions (Fig. 1C) demonstrated progressive enhancement of X-ray development with increasing nBM content, indicating the rising of scaffold density. Quantitative analysis of porosity (Fig. 1D) confirmed sustained high porosity exceeding 85% across all groups. The compression testing revealed significant mechanical reinforcement correlating with nBM incorporation (Fig. 1E). The PLGA/30nBM group exhibited greater compressive strength compared to PLGA and PLGA/10nBM groups (*p* < 0.05). Although the PLGA/30nBM group showed higher strength than the PLGA/20nBM group, this difference did not reach statistical significance. A clear dose-dependent strengthening effect was observed, with each incremental nBM addition producing measurable mechanical enhancement. In order to meet the requirements of bone tissue repair as far as possible, PLGA/30nBM was selected for subsequent experiments.

In TCM, deer bone is revered for its ability to fortify tendons and bones. And the nBM was abundant in active proteins, collagen, trace elements, and other beneficial components. [8] In Wu’s report, [11] a bone tissue engineering microsphere containing nBM was developed. It was confirmed that the addition of nBM significantly enhanced the osteogenic activity of the composite microspheres. These studies underscore the potential of nBM as a biomimetic matrix component for bone tissue engineering repair. By integrating nBM with biocompatible polymer materials, it is conceivable to enhance the repair efficacy of the composite materials. In this study, PLGA/nBM composite scaffolds were fabricated via an integrated phase inversion-particulate leaching methodology. The resultant interconnected macroporous architecture, characterized by pore dimensions of 200-400 μm, aligns with established osteoconductive requirements for bone regeneration [25, 26]. While acknowledging the inherent trade-off between elevated porosity and diminished mechanical performance, the biomimetic design of scaffold successfully emulates the organic-inorganic composite structure of natural cancellous bone. The porous architecture of scaffold, which was beneficial to osteoblast growth and nutrient exchange, positioned it as a promising biomimetic composite scaffold for bone defect repair [27, 28]. Mechanical characterization revealed significant content-dependent reinforcement of nBM, with the PLGA/30nBM scaffold demonstrating greater compression modulus.

### Fabrication of CIGF1&Nef@PLGA/nBM scaffold

**Fig. 2:**
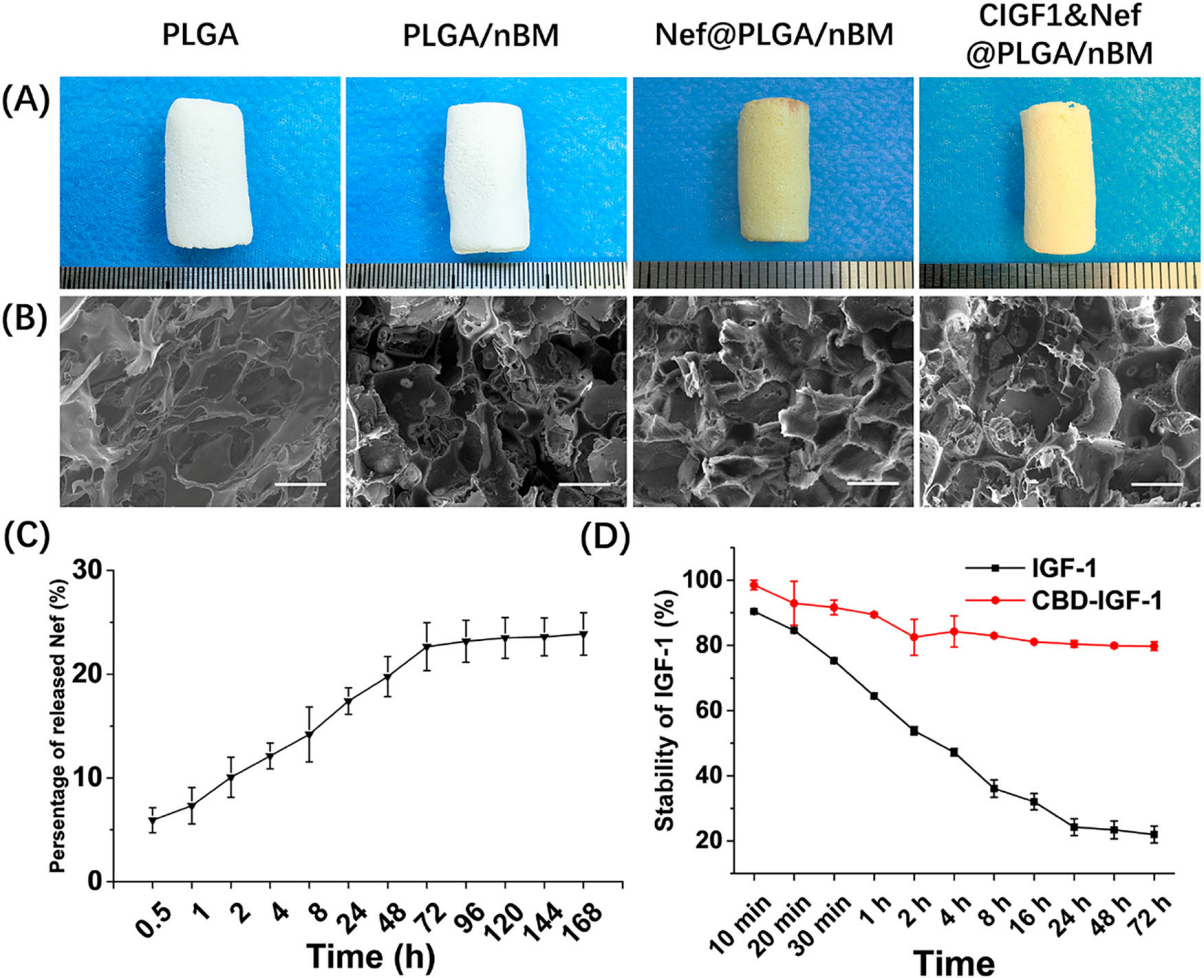
**A** the appearances and **B** representative SEM photos of scaffold from different groups. **C** The release behavior of Nef in 168 h. **D** The stability of IGF-1 and CBD-IGF-1 on scaffold. Bar = 200 μm, *n* = 3

According to the result shown in Fig. S1, the CBD-IGF-1 was successfully prepared. The expression level based on the 1 L expression system was approximately 0.5173 mg. According to SDS‑PAGE analysis, the protein purity was greater than 95%. The appearances of different scaffold (PLGA, PLGA/nBM, Nef@PLGA/nBM, and CIGF1&Nef@PLGA/nBM) were shown in Fig. 2A. While PLGA and PLGA/nBM groups maintained the characteristic white of native polymer, the Nef@PLGA/nBM and CIGF1&Nef@PLGA/nBM scaffolds were light yellow in color due to the Nef addition. SEM photos (Fig. 2B) confirmed preservation of the porous architecture across all modified scaffolds. Notably, the introduction of both CBD-IGF-1 and Nef did not significantly alter structural parameters of scaffold, demonstrating excellent compatibility between functional additives and the base matrix. FT‑IR spectra of the various scaffolds are presented in Fig. S5. The stretching vibration absorption peak of the phenyl group, indicative of Nef incorporation, was observed at approximately 2845 cm⁻¹ in both the Nef@PLGA/nBM and CIGF1&Nef@PLGA/nBM groups. The characteristic peak near 1652 cm⁻¹, corresponding to the amide group from nBM and CBD‑IGF‑1, was detected in all groups except the PLGA group. The peak around 560 cm⁻¹ was assigned to the P–O bending vibration, representing the inorganic phosphate phase in nBM. Furthermore, the characteristic absorption bands at 1597 cm⁻¹, 1032 cm⁻¹, and 827 cm⁻¹ were attributed to C = C, –C–O–C–, and –C–C–, respectively.

### Nef and CBD-IGF-1 release profiles

Based on the EE and LC of Nef in scaffolds (Table S2 & S3), the release behavior of Nef in 168 h was calculated and shown in Fig. 2C. It exhibited an initial fast release of Nef within the first 72 hours, which was determined to be 22.65 ± 2.30%. Then, the Nef showed sustained release. The cumulative release of Nef reached 23.88 ± 2.04% and was still continuing to be released slowly. The adhesion and release of CBD-IGF-1 were quantitatively detected via the ELIZA method. According to the result in Table S4, the adhesion rate of CBD-IGF-1 on scaffold was 84.23 ± 4.12%, while that of IGF-1 was 51.95 ± 2.83%. The adhesion ability of CBD-IGF-1 to biomimetic scaffold was significantly higher than that of IGF-1 (*p* < 0.05). As shown in Fig. 2D, CBD-IGF-1 on scaffold was released at an extremely slow rate over time. After 72 h, 79.98 ± 1.33% of CBD-IGF-1 remained on the surface of scaffold. At the same time, IGF-1 showed rapid release from scaffold. After 72 h, only 21.96 ± 2.56% of IGF-1 remained.

### Cytocompatibility of CIGF1&Nef@PLGA/nBM scaffold

**Fig. 3:**
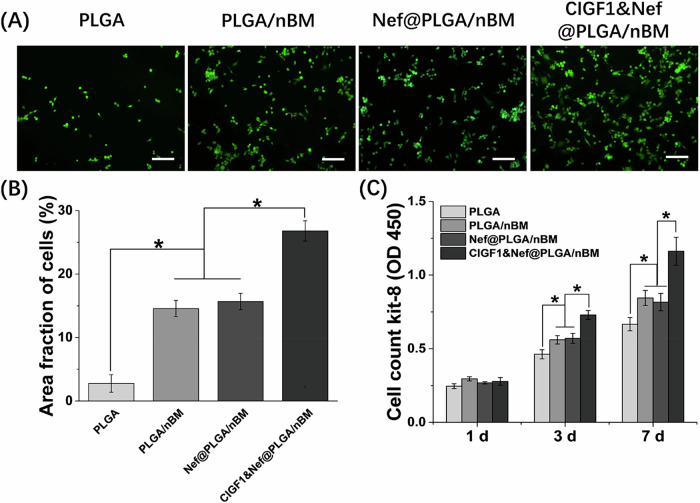
Cytocompatibility of different scaffolds. **A** Calcein-AM staining photos and **B** area fraction of MC3T3-E1 cells on scaffold in each group. **C** proliferation of MC3T3-E1 cells at different time points (1, 3, and 7 days). Bar = 200 μm, *n* = 3, *p < 0.05

On the 3rd day of culture, the adhesion and growth of MC3T3-E1 cells on the surface of each group of materials was determined by Calcein-AM staining. As shown in Fig. 3A, living cells on the surface of the material were labeled by green fluorescence. And the area fraction of cells in each group was revealed in Fig. 3B. Compared with PLGA group, the cell adhesion on the surface of PLGA/nBM and Nef@PLGA/nBM groups was significantly increased (*p* < 0.05). The amount of cells adhered on CIGF1&Nef@PLGA/nBM material was the highest, and significantly higher than that in other groups (*p* < 0.05). Cell proliferation at different time points was shown in Fig. 3C. The trend of cell proliferation in each group was consistent with the cell adhesion results. on the 3rd and 7th days, compared with PLGA group, the cell proliferation of PLGA/nBM and Nef@PLGA/nBM groups was significantly accelerated (*p* < 0.05). The cell proliferation of CIGF1&Nef@PLGA/nBM group was the highest, and significantly higher than that in other groups (*p* < 0.05). These results fully indicated that both nBM and IGF-1 in the scaffold could promote the adhesion and proliferation of MC3T3-E1 cells on the material.

### Osteo-differentiation promotion of CIGF1&Nef@PLGA/nBM scaffold

**Fig. 4:**
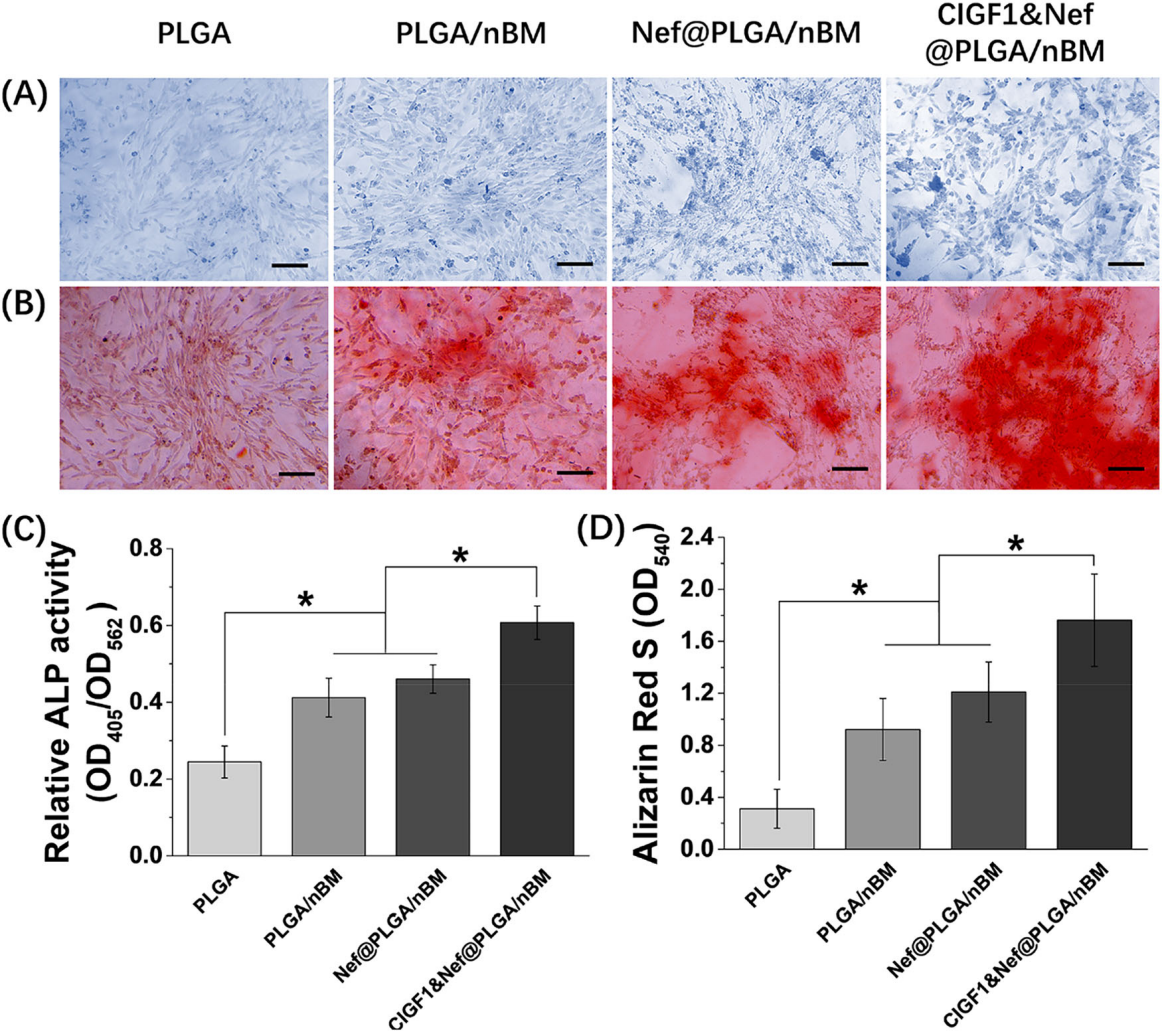
Osteo-differentiation promotion of different scaffolds. **A** ALP staining photos of MC3T3-E1 cells on scaffold in each group at 7th day. **B** ARS staining photos of MC3T3-E1 cells on scaffold in each group at 14th day. **C** the relative ALP activity of MC3T3-E1 cells on scaffold in each group at 7th day. **D** the quantitation results of ARS at 14th day. Bar = 100 μm, *n* = 3, *p < 0.05

ALP activity is an important marker to evaluate osteoblast differentiation. As shown in Fig. 4A, the ALP staining in PLGA group was inconspicuous. Compared with PLGA group, ALP staining was more obvious in PLGA/nBM and Nef@PLGA/nBM groups. The ALP staining in CIGF1&Nef@PLGA/nBM group was most evident. A large amount of dark blue staining can be observed in the field of view. The results of relative ALP activity (Fig. 4C) were consistent with those of ALP staining. At the 7th days, compared with PLGA group, the relative ALP activity of PLGA/nBM and Nef@PLGA/nBM groups was significantly accelerated (*p* < 0.05). The relative ALP activity of CIGF1&Nef@PLGA/nBM group was the highest, and significantly higher than that in other groups (*p* < 0.05). Nef@PLGA/nBM group had higher ALP activity than PLGA/nBM group. however, the difference was not significant. The ARS staining and quantitation results at 14 days were respectively revealed in Fig. 4B, D. The calcium nodules produced by osteoblast were stained red by ARS. As shown in the photos, the red-staining was most obvious in CIGF1&Nef@PLGA/nBM group and most inconspicuous in PLGA group. The quantitative results proved the significance of the difference between these two groups with other groups (*p* < 0.05). In Nef@ PLGA/nBM group, the ARS staining was slightly more obvious than that in PLGA/nBM group. However, the quantitative results showed that there was no significant difference between them.

### Expression of osteogenic genes and proteins

**Fig. 5:**
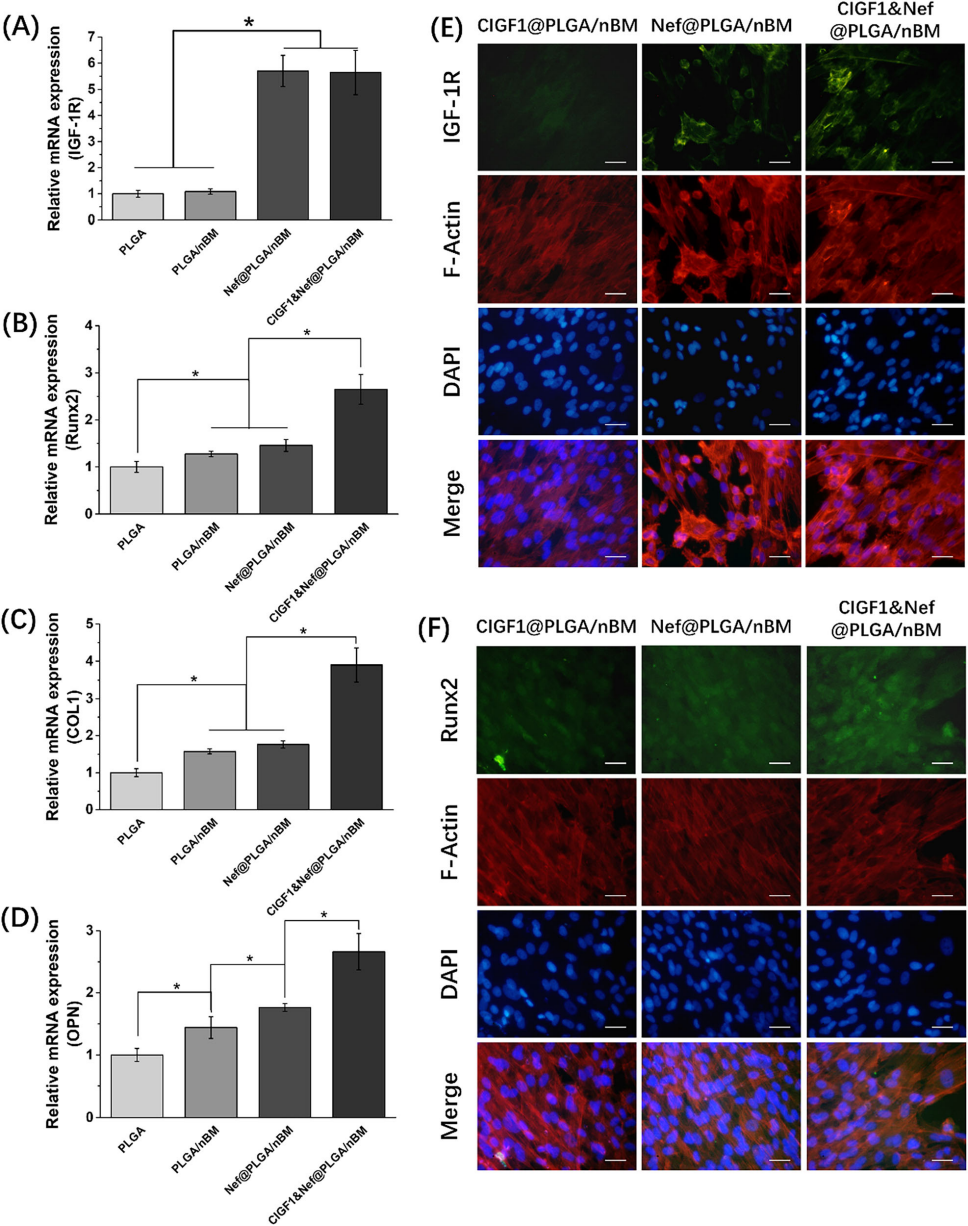
Expression of osteogenic genes and proteins. The relative mRNA expression of **A** IGF-1R, **B** Runx2, **C** COL1, and **D** OPN in MC3T3-E1 cells on different scaffolds at 7th day. The immunofluorescence staining photos of **E** IGF-1R and **F** Runx2 in MC3T3-E1 cells on different scaffolds at 7th day. Bar = 50 μm, *n* = 3, *p < 0.05

The results of RT-PCR were shown in Fig. 5A–D. The research result showed that the mRNA expression of IGF-1R in Nef containing groups was remarkably accelerated in comparison with that in PLGA and PLGA/mBM groups (*p* < 0.05). Interestingly, mRNA expression of IGF-1R was not higher in the IGF-1-containing CIGF1&Nef@PLGA/nBM group than in the Nef@PLGA/nBM group. It was suggested that IGF-1R expression promoted by Nef was sufficient for interaction with IGF-1. There was synergy between Nef and IGF-1.

RUNX2 is one of the key transcription factors in osteoblast differentiation and is highly expressed in osteoblast differentiation. As shown in Fig. 5B, the mRNA expression of Runx2 was highest in CIGF1&Nef@PLGA/nBM group and lowest in PLGA group. The results proved the significance of the difference between these two groups with other groups (*p* < 0.05). The nBM could upregulated the mRNA expression of Runx2, further enhanced the osteogenic differentiation. In coordination with Nef, the expression of Runx2 promoted by IGF-1 was upregulated to more than twice that of PLGA/nBM group. The CIGF1&Nef@PLGA/nBM scaffold showed strong ability to promote osteoblast differentiation at the molecular biological level. COL1 gene expression represents an increase in cell maturity, and COL1 is highly expressed in the bone matrix. OPN is an important component of the bone matrix and is highly expressed in the middle stage of osteogenic differentiation. According to the results, the mRNA expression of COL1 and OPN was upregulated eminently in CIGF1&Nef@PLGA/nBM group (*p* < 0.05). The nBM, Nef, and IGF-1 in scaffold together enhanced the expression of bone matrix protein in osteoblasts at the molecular level.

In order to confirm the synergistic effect of Nef and IGF-1 on osteogenic differentiation, immunofluorescence staining was employed to demonstrate the effects of three different scaffolds (CIGF1@PLGA/nBM, Nef@PLGA/nBM, and CIGF1&Nef@PLGA/nBM) on the expression of IGF-1R and Runx2 proteins in cells. The immunofluorescence staining photos were presented in Fig. 5E, F. And the quantitative analysis of the immunofluorescence staining results was shown in Fig. S6. As shown in Fig. 5E, the stained IGF-1R protein in Nef containing groups was much more obvious in comparison with that in CIGF1@PLGA/nBM group. And the green dyeing of IGF-1R was not more remarkable in the CIGF1&Nef@PLGA/nBM group than in the Nef@PLGA/nBM group. The immunofluorescence staining of Runx2 was observed in Fig. 5F. The stained Runx2 in CIGF1&Nef@PLGA/nBM group was much more obvious than that in CIGF1@PLGA/nBM and Nef@PLGA/nBM groups. IGF-1 and Nef synergistically promoted Runx2 expression and subsequent osteogenic differentiation. These results confirmed a potential synergistic osteogenesis-promoting mechanism of Nef and IGF-1 in CIGF1&Nef@PLGA/nBM scaffold. The Nef in composite scaffold was first effectively activating the expression of IGF-1R, thereby enhancing IGF-1-mediated osteogenic differentiation in the presence of IGF-1.

### Repair effect of skull injury

**Fig. 6:**
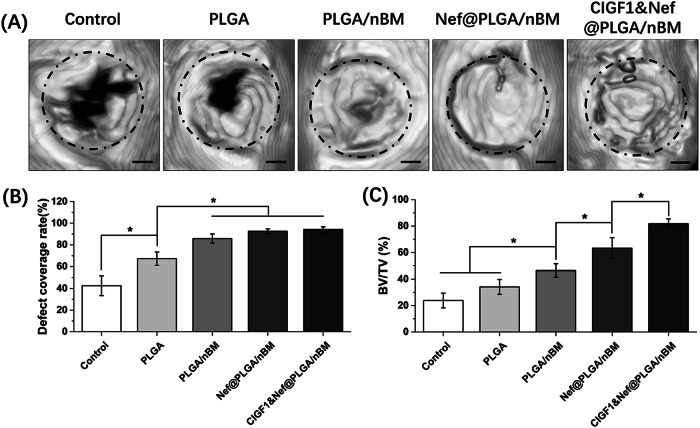
The micro-CT results of specimens at 8 weeks postoperatively. **A** representative 3D model, **B** defect coverage rate, and **C** bone volume fraction (BV/TV) of each sample. Bar = 1 mm, *n* = 3, *p < 0.05

At 8 weeks postoperatively, the animals were sacrificed. The complete skull bone was removed. All samples were scanned by micro-CT. 3D modeling was performed to observe the formation of new bone. The representative models were shown in Fig. 6A. And the defect coverage rate was calculated revealed in Fig. 6B. The results showed that the bone defects in control group and PLGA group were still not completely closed. While the bone defects in other three groups had been almost completely closed by new bone. Bone volume fraction (BV/TV) of each sample was analyzed via CTan software and displayed in Fig. 6C. The bone volume fraction of CIGF1&Nef@PLGA/nBM group was outstanding higher than other groups (*p* < 0.05). CIGF1&Nef@PLGA/nBM scaffold demonstrated great potential in promoting new bone formation.

**Fig. 7:**
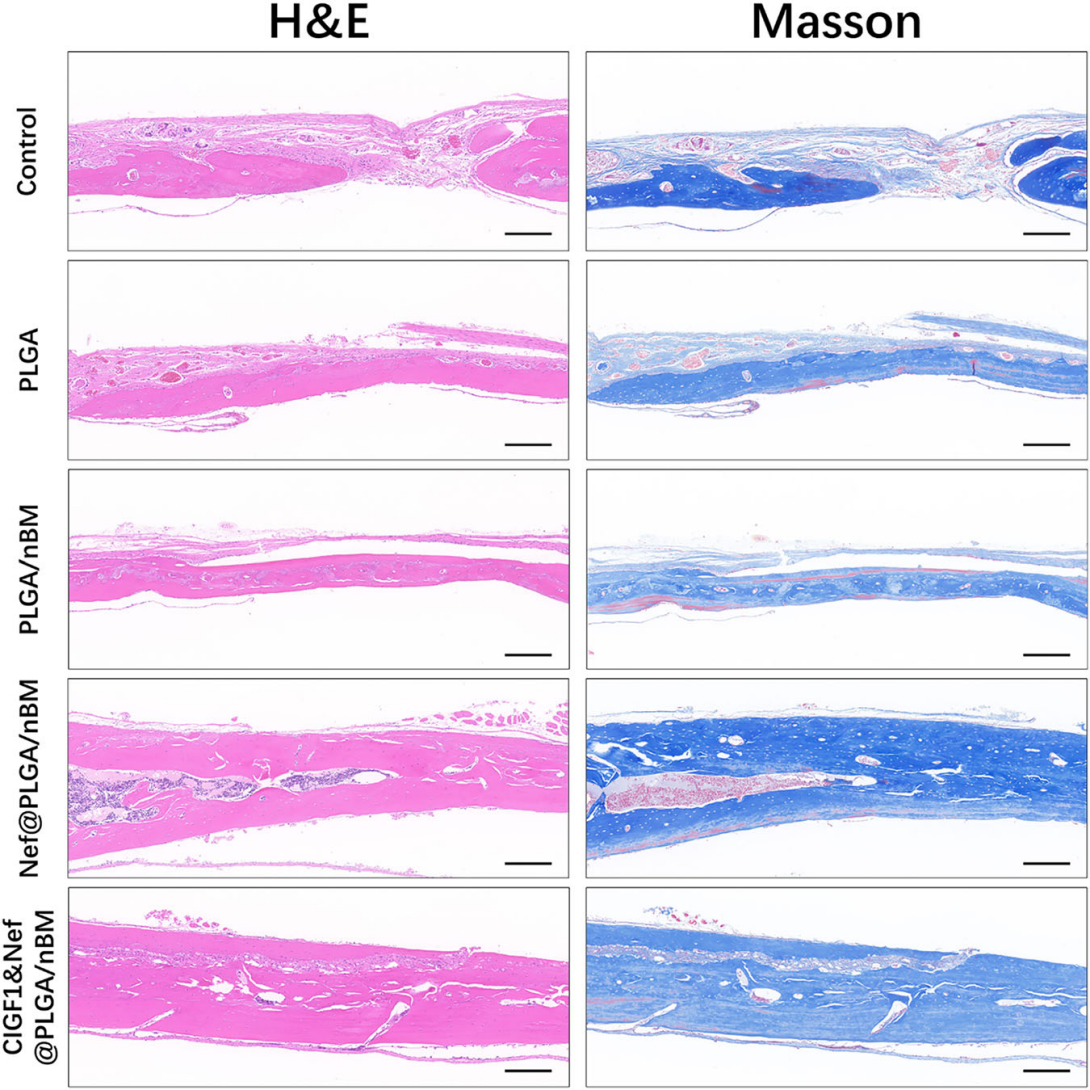
The photos of H&E and Masson’s trichrome staining of specimens at 8 weeks postoperatively. Bar = 100 μm

Histological evaluation through H&E and Masson’s trichrome staining (Fig. 7) revealed distinct regeneration across experimental groups. H&E sections illustrated progressive osteointegration, with the composite scaffold functioning as an osteoconductive bridge facilitating neo bone formation. Notably, the CIGF1&Nef@PLGA/nBM group exhibited hypertrophic bone callus formation.

The result of Masson staining indicated that most of the defect sites in each group had been covered with newborn tissue. Scattered mature bone tissue stained red can also be observed inside the blue new bone. The thickness of new bone in the CIGF1&Nef@PLGA/nBM group was thicker than that in the other groups. There was also more red staining in the new bone site of this group. These results further confirmed that CIGF1&Nef@PLGA/nBM could enhance the effect of bone defect repair.

## Discussions

Currently, bone-derived materials are widely employed in bone repair applications. Among these, bones from sika deer and red deer are particularly rich in bioactive proteins, collagen, trace elements, and other biologically functional components. In the context of TCM, deer bone has long been recognized for its efficacy in strengthening tendons and bones [8]. Previous studies have demonstrated that polypeptide fractions extracted from Sika deer bone can improve the pathological microstructure of osteoporotic bone and upregulate eNOS expression in osteoblasts [9]. however, research investigating the use of deer bone for bone tissue engineering regeneration is still scarce. In Wu’s report, a bone tissue engineering microsphere containing nBM was developed. It was confirmed that the addition of nBM significantly enhanced the osteogenic activity of the composite microspheres [11]. These studies underscore the potential of nBM as a biomimetic matrix component for bone tissue engineering repair. By integrating nBM with biocompatible polymer materials, it is conceivable to enhance the repair efficacy of the composite materials. In this study, PLGA/nBM composite scaffolds were fabricated via an integrated phase inversion-particulate leaching methodology. The resultant interconnected macroporous architecture, characterized by pore dimensions of 200–400 μm, aligns with established osteoconductive requirements for bone regeneration [25, 26]. Porous scaffolds play a critical role in successful in vivo bone regeneration. Compared with non‑porous PLGA scaffolds, porous structures provide sufficient space for cell infiltration, proliferation, nutrient diffusion, and tissue ingrowth [29]. They also facilitate vascularization and allow mechanical integration between the scaffold and the host bone tissue [30, 31]. In contrast, non‑porous PLGA scaffolds lack internal channels, which severely limits cell migration, nutrient transport, and bone tissue ingrowth, resulting in poor osteointegration and unsatisfactory bone regeneration. Therefore, the porous structure is essential for enhancing bone repair efficiency. Although non-porous scaffolds exhibit certain advantages in mechanical properties compared with porous scaffolds, the mechanical performance of porous scaffolds can be effectively enhanced by incorporating inorganic components, thereby meeting the mechanical requirements for bone tissue repair. In the present study, while acknowledging the inherent trade-off between elevated porosity and diminished mechanical performance, the biomimetic design of scaffold successfully emulates the organic-inorganic composite structure of natural cancellous bone. The porous architecture of scaffold, which was beneficial to osteoblast growth and nutrient exchange, positioned it as a promising biomimetic composite scaffold for bone defect repair [27, 28].

The degradation rate of the material depends not only on the molecular weight and feed ratio of the raw materials, but also on the processing technology, material topography and microenvironment [32, 33]. The molecular weight of PLGA raw materials decrease during the dissolution and phase inversion processes [34]. In addition, the scaffolds fabricated via the method established in this study possessed hierarchical microstructures, including micron-scale pores, micro/nano-scale pores and surface grooves. These synergistic factors facilitated the infiltration of bodily fluids into the scaffolds following in vivo implantation. And the scaffolds further acted in concert with various enzymes in the bodily fluids to accelerate the degradation rate [33]. Although complete degradation was not achieved, the overall structural integrity of the scaffolds was compromised, thus precluding any impediment to repair. The findings of this study further verify the excellent compatibility between scaffold degradation and bone growth. Mechanical characterization revealed significant content-dependent reinforcement of nBM, with the PLGA/30nBM scaffold demonstrating greater compression modulus. In order to meet the requirements of bone tissue repair as far as possible, PLGA/30nBM was selected for subsequent experiments.

Although Nef has been proved to be an effective osteogenic active agent, high doses of Nef have been reported to inhibit cell growth [17]. The sustained local release profile of Nef within the bone defect served dual advantages of osteo-induction and biosafety [35]. Porous scaffolds prepared by particle leaching can be used as sustained drug release carriers [36, 37]. Recent investigations into the osteogenic optimization of PLGA-based scaffolds have demonstrated concentration-dependent biological effects of Nef. As evidenced by Wu et al., the maximal osteogenic enhancement MC3T3-E1 cell was achieved at 25 μM Nef concentration [17]. According to this finding, the present study standardized Nef incorporation at 25 μM within the PLGA solution. In the present study, it exhibited a stable release of Nef from scaffold. This effectively reconciled the osteogenic potential with dose-dependent cytotoxic of Nef.

IGF-1 is a growth factor that exhibits prominent effects in bone injury repair, promoting both the proliferation and differentiation of osteoblasts. However, IGF-1 has a short half-life and is prone to being attenuated with body fluid, which weakens its therapeutic efficacy in vivo. To overcome these limitations, the development of bioengineered drug delivery systems capable of stably loading and releasing IGF-1 has attracted a lot of interest [18, 19]. Growth factors equipped with binding tags provided through recombinant expression and ligation techniques demonstrate significant potential in tissue engineering applications. The binding domains derived from antibodies, enzymes, and other biomacromolecules play a crucial role in the integration of growth factors with biomaterials [20]. There is a large amount of collagen matrix in nBM. The growth factors with CBD have been lucubrated as effective agents binding specially to collagen material [22]. To biofunctionalized scaffold surface, CBD-IGF-1 was implemented in this research. The CBD (TKKTLRT) was derived from mammalian collagenase [38]. The CBD-IGF-1 targeting approach to collagen matrix within nBM through orthogonal bioconjugation, enabling binding of growth factors on scaffold. The results indicated that the adhesion ability of CBD-IGF-1 to biomimetic scaffold was significantly higher than that of IGF-1. And the CBD-IGF-1 on scaffold was released at an extremely slow rate over time. CBD assisted the stable binding and release of IGF-1 on the scaffold surface. This was conducive to IGF-1 to play a long-term role in the bone defect site.

During the implantation, osteoblasts recruited from the surrounding tissue must first adhere to the scaffold surface and colonize to proceed the subsequent bone repair process [25]. Collagen is a main component of bone matrix and mediated cell adhesion and growth [21]. IGF-1 is a growth factor that exhibits prominent effects in promoting proliferation of osteoblasts [19]. The results of this study indicate that CIGF1&Nef@PLGA/nBM scaffold can effectively promote the adhesion and proliferation of osteoblasts on the scaffold surface, which may be related to the collagen and IGF-1 in scaffold. This conclusion provided an important basis for the application of CIGF1&Nef@PLGA/nBM scaffold in bone repair.

The ability to induce osteogenic differentiation is important for evaluating the biological activity of bone repair materials. Levels of ALP activity can be used to characterize the early stage of osteogenic differentiation of cells. And the cellular calcium deposition can be used as a late marker of osteoblastic differentiation. Relevant research has illuminated that the nBM can moderate the micro-structural abnormalities in osteoporosis[10, 39] and enhanced the osteogenic activity of the bone tissue engineering materials [11]. The results in this study confirmed the osteogenic activity of nBM. Nef is another known osteo-inductive agent from TCM to exert osteogenesis through IGF-1R-related pathways [16, 17]. Unexpectedly, the Nef in scaffold did not exhibit outstanding osteogenic activity. In Wu’s report, although the Nef alone could induce osteogenic differentiation of MC3T3-E1 cells, it achieved obviously osteogenesis through the synergistic action of IGF-1 induced by bioactive glass [17]. In the present study, the osteogenesis activity of Nef might be masked by nBM. However, the addition of IGF-1, another growth closely associated with osteogenesis, further enhanced the osteogenic activity of the scaffold. These results further provided an important basis for the application of CIGF1&Nef@PLGA/nBM scaffold in bone repair.

In the process of bone tissue regeneration, various behaviors of cells are regulated by different genes. IGF-1R is a key receptor for IGF-1-mediated osteogenic differentiation in IGF-1R/PI3K/AKT/mTOR pathway [17, 40]. Nef has recently emerged as a viable therapeutic agent for combating osteolytic bone conditions, including but not limited to osteoporosis. Wu et al. prepared a bone tissue engineering scaffold containing Nef for bone injury repair. The composite scaffold containing Nef was first shown to effectively activate the IGF-1R/PI3K/AKT/mTOR pathway, thereby enhancing IGF-1-mediated osteogenic differentiation [17]. Building on the findings of this report, IGF-1 embedded within the scaffold material further triggered the activation of IGF-1R-mediated signaling cascades, while the synergistic action of IGF-1 and Nef in promoting bone formation was elucidated for the first time. This approach enabled the CIGF1&Nef@PLGA/nBM scaffold to further activate RUNX2 expression, enhance cellular osteogenic differentiation, and stimulate the production of key osteogenic matrix proteins (COL1 and OPN). These results delineate the potential mechanism underlying the CIGF1&Nef@PLGA/nBM scaffold’s efficacy in skull injury repair, offering a theoretical reference for the optimization of related therapeutic schemes.

## Conclusion

In this research, a novel biomimetic bone scaffold functionalized with two TCM ingredients (nBM and Nef) and collagen-binding IGF-1 was fabricated. The biomimetic CIGF1&Nef@PLGA/nBM scaffold mimicked cancellous bone in porous structure and bone matrix composition rich in collagen. Both Nef contained in the scaffold and CBD-IGF-1 bound to collagen matrix could be slowly released from the scaffold to play a long-term and safety bioactivity. In vitro tests showed that the biomimetic CIGF1&Nef@PLGA/nBM scaffold had good cytocompatibility and osteogenic activity. Nef and IGF-1 play a synergistic role in IGF-1-mediated osteogenic differentiation via IGF-1R axis to ensure the osteogenic activity of the biomimetic scaffold. In vivo skull defect repair experiment confirmed that CIGF1&Nef@PLGA/nBM could enhance the neocortical bone formation and osseous maturation, further accelerated the bone defect repair. The findings of this research demonstrate that the biomimetic CIGF1&Nef@PLGA/nBM scaffold is a promising implantation in bone defect therapy, and will provide a new research direction for the application of TCM ingredients in bone tissue engineering.

## Supplementary information

Supplementary information
