# Supplementary material for: Biomimetic bone scaffold functionalized with traditional Chinese medicine ingredients and collagen-binding IGF-1 for bone regeneration
Source: J Mater Sci Mater Med. 2026 Apr 6;37(1):62. doi: 10.1007/s10856-026-07041-2 (PMC13219079; doi:10.1007/s10856-026-07041-2)
Supplement: Supplementary file 1 — Supplementary information [file 10856_2026_7041_MOESM1_ESM.docx]

**Supplementary Material**

**Biomimetic bone scaffold functionalized with traditional Chinese medicine ingredients and collagen-binding IGF-1 for bone regeneration**

Xiaobo Gao^1,2^, Xiaomin Zhao^2^, Ying Zhang^2^, Bocheng Lei ^3,^*

^1^ Department of Stomatology, Chifeng municipal hospital, Chifeng, Inner Mongolia, 024000, P.R. China.

^2^ Affiliated Chifeng Clinical Medical College of Inner Mongolia Medical University, Chifeng, Inner Mongolia, 024000, P.R. China.

^3^ Stomatology College of Chifeng University, Chifeng, Inner Mongolia, 024000, P.R. China.

* Correspondence: Bocheng Lei, email: 1178609705@qq.com

**
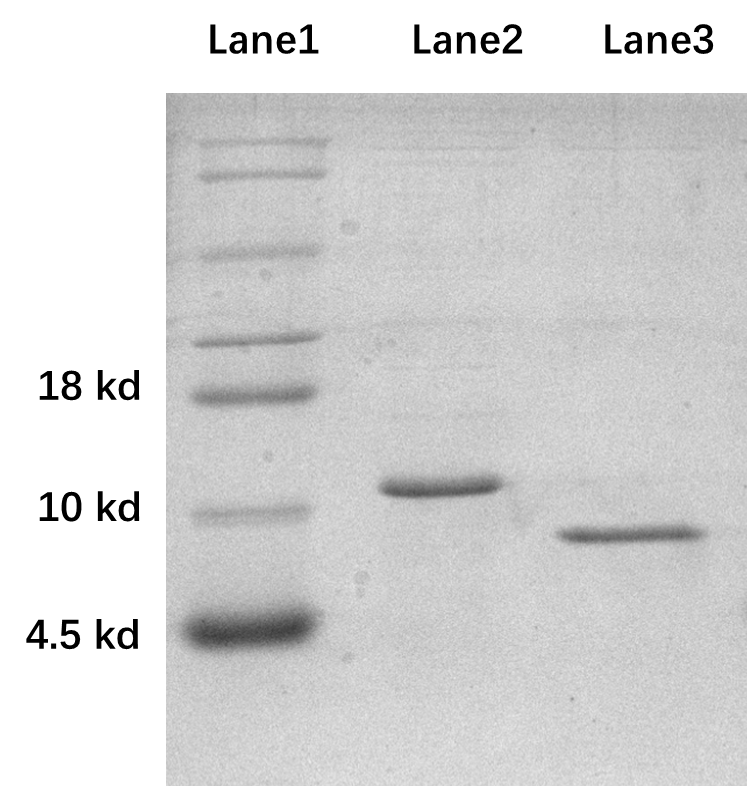
**

**Figure S1** the SDS-PAGE result of recombinant IGF-1. Line 1: marker; lane 2: CBD-IGF-1; lane 3: commercial IGF-1.

**
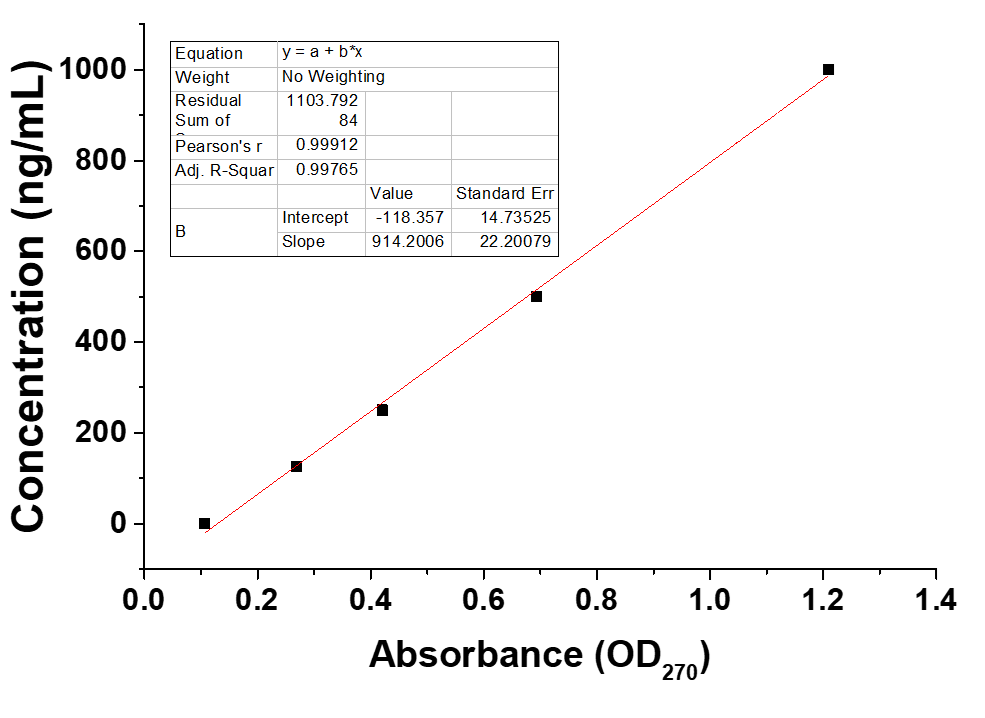
Figure S2.** Standard calibration curve established using methanolic solutions containing known Nef concentrations through UV spectrophotometric analysis at a detection wavelength of 270 nm.

**
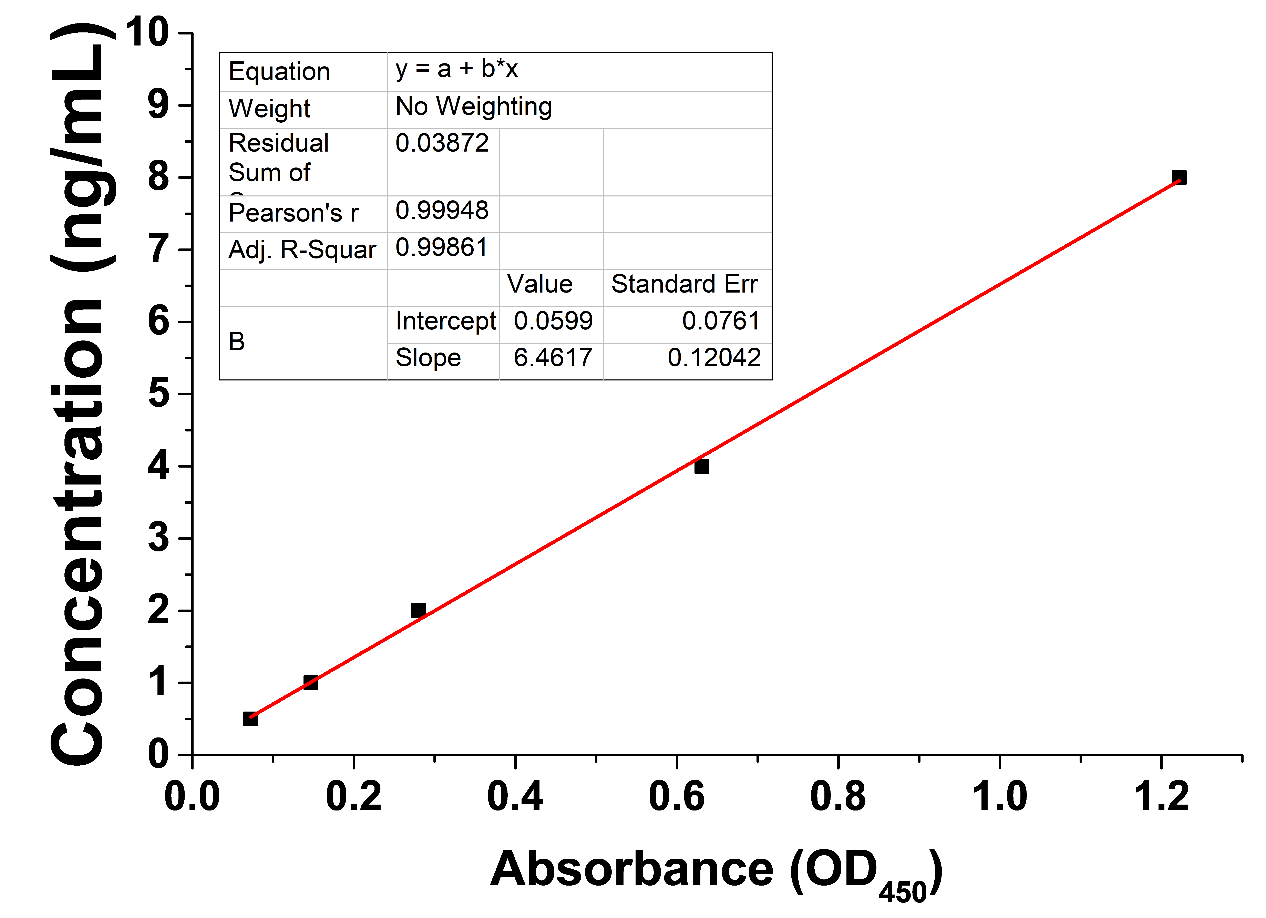
Figure S3.** Standard calibration curve of the IGF-1 ELISA kit.

**
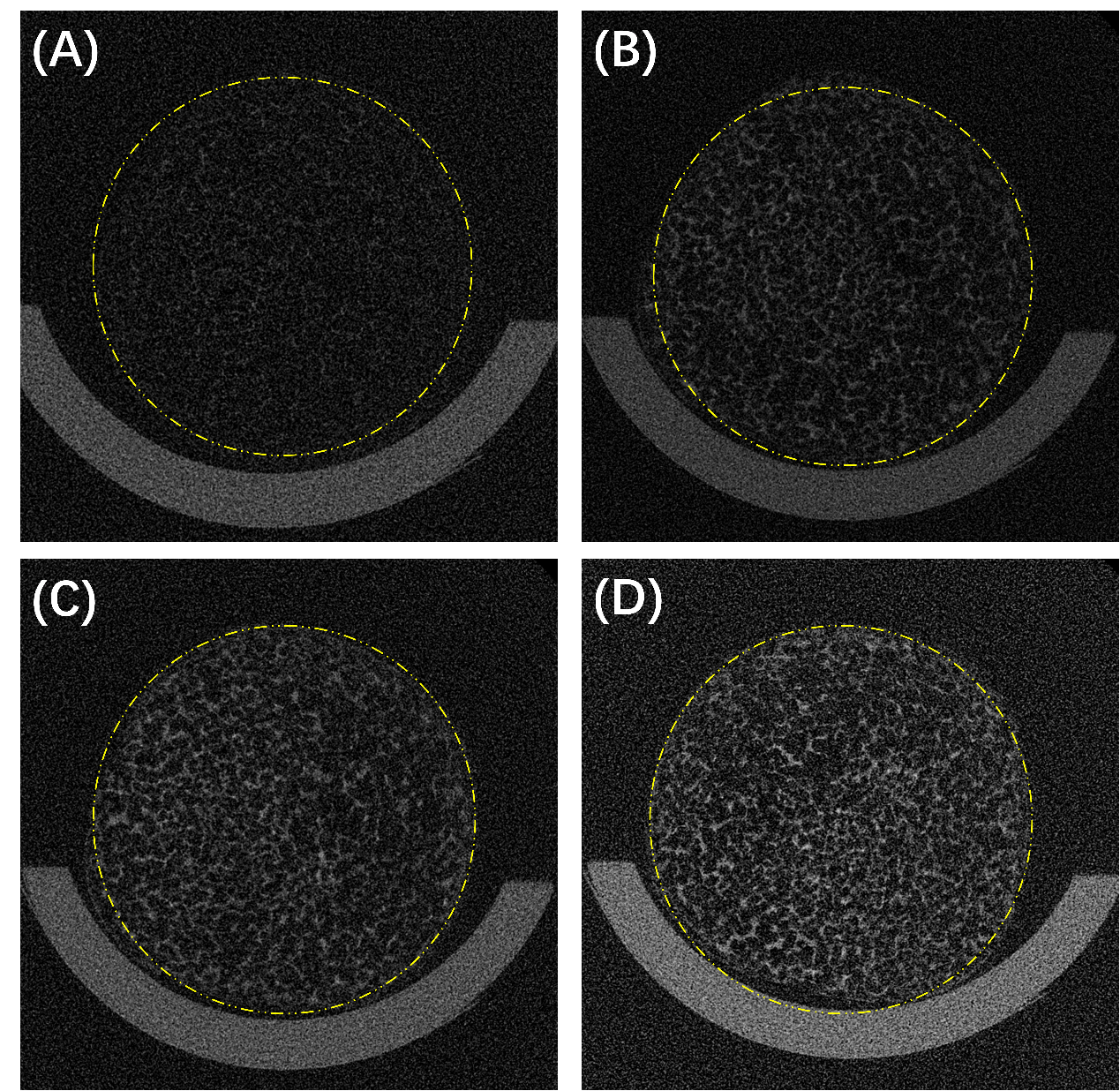
Figure S4**. Cross-sectional X-ray images of the scaffolds obtained by MicroCT. (A)PLGA; (B)PLGA/10nBM; (C) PLGA/20nBM;(D) PLGA/30nBM.

**Figure S5.** Analysis of the FT-IR spectra of scaffolds in each group.

**Figure S6.** The quantitative analysis of the immunofluorescence staining results.

**Table S1.** Primers used in RT-PCR

| **Gene** | **Forward primer sequence (5’-3’)** | **Reverse primer sequence (3’-5’)** |
| --- | --- | --- |
| **COL1** | CCCAGCGGTGGTTATGACTT | TCGATCCAGTACTCTCCGCT |
| **Runx2** | GCCAGTAATCTTCGTGCCAG | TAGTGAGCTTCTTCCTGGGGA |
| **OPN** | CCAGCCAAGGACCAACTACA | AGTGTTTGCTGTAATGCGCC |
| **IGF-1R** | TTTCCACTCCGCATTTCTGC | CGTCCAAAAACAAGAGCGCA |
| **GAPDH** | CTTGTGCAGTGCCAGCCTC | GATGGTGATGGGTTTCCCGT |

**Table S2.** Information of primary antibodies in immunofluorescence staining

| **Name** | **Brand** | **Product code** | **Host species** | **Dilution ratio** |
| --- | --- | --- | --- | --- |
| Runx2 | Invitrogen | MA5-32373 | Rabbit | 1: 100 |
| IGF-1R | Invitrogen | PA5-79444 | Rabbit | 1: 100 |

**Table S3.** EE and LC of Nef in scaffolds (100 mg)

|  | EE (%) | LC (%) |
| --- | --- | --- |
| Nef | 73.07 ± 3.19 | 0.0057 ± 0.0015 |

**Table S4.** Adhesion amount of IGF-1 and CDB-IGF-1 on scaffolds (100 mg)

|  | Adhesion amount (ng) |
| --- | --- |
| IGF-1 | 51.95 ± 2.83 ng |
| CBD-IGF-1 | 84.23 ± 4.12 ng |
